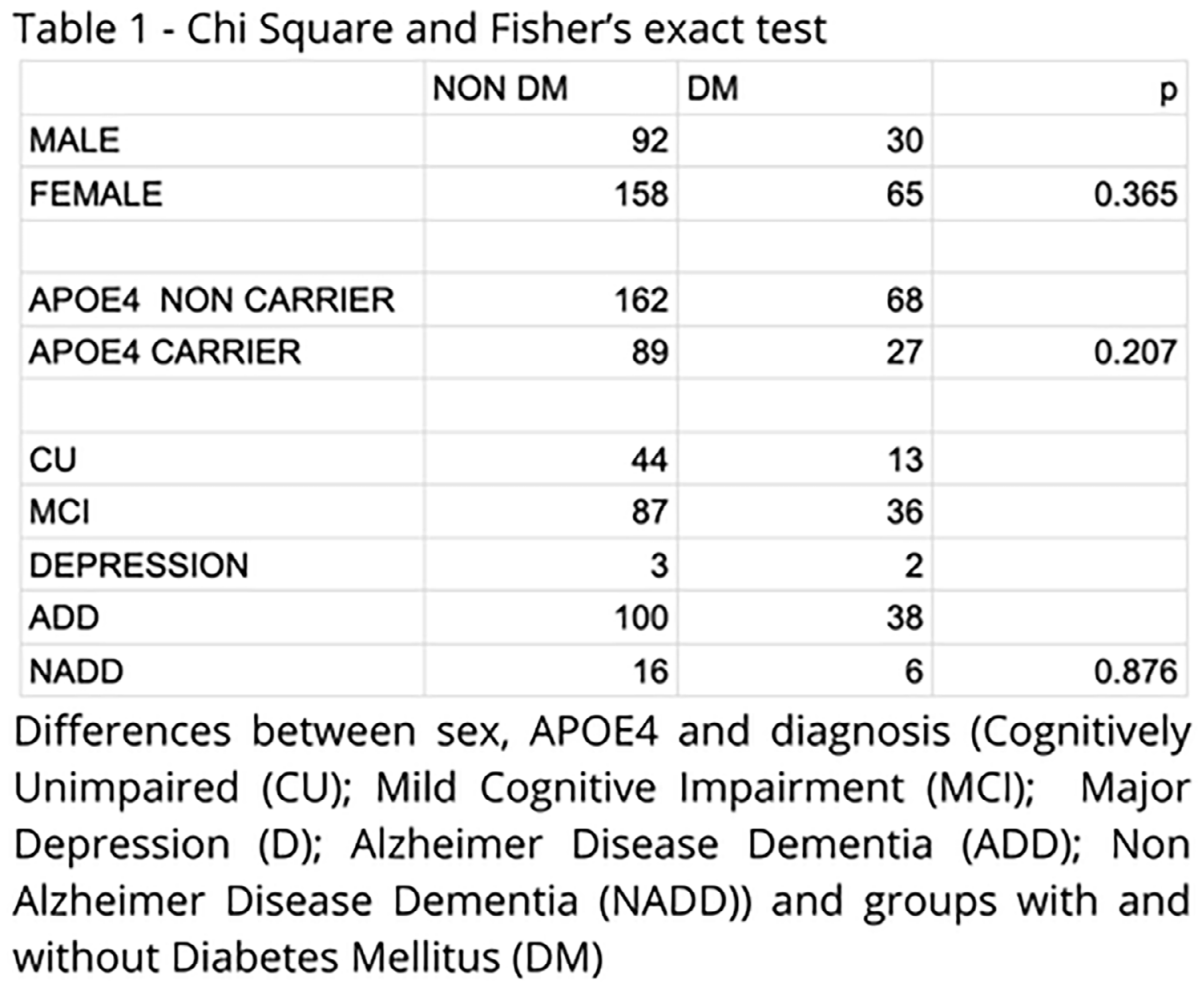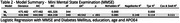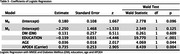# Diabetes Mellitus influences on cognitive abilities of older adults compared to others risks factors

**DOI:** 10.1002/alz70860_099929

**Published:** 2025-12-23

**Authors:** João Marcos Silva Borges, João Henrique Fonseca, Thaise Vallesca Queiroz, Flávia Carolina Lima Torres, Leonardo Ryuiti Kimoto, Giovanna Correia Pereira Moro, Marcelle Ferreira Saldanha, Gabriela Tomé Oliveira Engelmann, Cleidiney Alves e Silva, Marco Aurelio Romano‐Silva, Debora Marques de Miranda, Jonas Jardim de Paula, Bernardo de Mattos Viana, Maria Aparecida Camargos Bicalho

**Affiliations:** ^1^ Federal University of Minas Gerais, Belo Horizonte, Minas Gerais, Brazil; ^2^ Cog‐Aging Research Group, Universidade Federal de Minas Gerais (UFMG), Belo Horizonte, Minas Gerais, Brazil; ^3^ Cog‐Aging Research Group, Brazil, Belo Horizonte, Minas Gerais, Brazil; ^4^ Cog‐Aging Research Group, Belo Horizonte, Minas Gerais, Brazil; ^5^ Undergraduate Medicine, Faculty of Medicine, Universidade Federal de Minas Gerais (UFMG), Belo Horizonte, Minas Gerais, Brazil; ^6^ Older Adult's Psychiatry and Psychology Extension Program (PROEPSI), School of Medicine, Universidade Federal de Minas Gerais (UFMG), Belo Horizonte, Minas Gerais, Brazil; ^7^ Sciences Applied to Adult Health Postgraduate Program, School of Medicine, Universidade Federal de Minas Gerais (UFMG), Belo Horizonte, Minas Gerais, Brazil; ^8^ Molecular Medicine Program, School of Medicine, Federal University of Minas Gerais, Belo Horizonte, Minas Gerais, Brazil; ^9^ Older Adult's Psychiatry and Psychology Extension Program (PROEPSI), School of Medicine, Federal University of Minas Gerais, Belo Horizonte, Minas Gerais, Brazil; ^10^ Neurotec R National Institute of Science and Technology (INCT‐Neurotec R), Faculty of Medicine, Federal University of Minas Gerais, Belo Horizonte, Minas Gerais, Brazil; ^11^ Neurotec R National Institute of Science and Technology (INCT‐Neurotec R), Faculty of Medicine, Universidade Federal de Minas Gerais (UFMG), Belo Horizonte, Minas Gerais, Brazil; ^12^ Molecular Medicine Postgraduate Program, School of Medicine, Universidade Federal de Minas Gerais (UFMG), Belo Horizonte, Minas Gerais, Brazil; ^13^ Department of Psychiatry, School of Medicine, Federal University of Minas Gerais, Belo Horizonte, Minas Gerais, Brazil; ^14^ Geriatrics and Gerontology Center Clinical Hospital of University of Minas Gerais, Belo Horizonte, Minas Gerais, Brazil; ^15^ Department of Internal Medicine, School of Medicine, Federal University of Minas gerais, Belo Horizonte, Minas Gerais, Brazil

## Abstract

**Background:**

Diabetes Mellitus (DM) is an important risk factor for cognitive impairment and dementia. However, APOE4 allele and education are also strongly correlated to cognitive impairment and dementia diagnosis, and they may moderate the impact of DM on them.

Objective: To evaluate the association between DM and cognitive performance of older adults from different cognitive profiles, comparing with education, sex, age and APOE4 carrier.

**Methods:**

This is a cross‐sectional study nested on the Cog‐Aging cohort study, which was previously approved by the local ethics committee. A total of 345 participants were selected with different cognitive diagnosis: 57 Cognitively Unimpaired (CU); 123 Mild Cognitive Impairment (MCI); 5 Major Depression (MD); and 160 Mild Dementia (D). Age, education, sex, APOE4 carrier status and total performance on Mini Mental State Examination (MMSE) were collected. The 19‐20 cuttoff for illiterate and 23‐24 for literate was used to evaluate cognitive ability. Chi‐Square and Fisher's exact test were used to assess the differences in sex, APOE4 and diagnosis in groups with and without DM. Logistic regression was applied to analyse MMSE cutoff points and APOE4, DM, education and age.

**Results:**

DM was observed in 95 participants (27.53%) and 116 (33.62%) were APOE4 carriers. There was no difference between sex, APOE4, and cognitive groups and DM (Table 1). By logistic regression, age (*p* = 0.044), education (*p* <0.001) and APOE4 (*p* = 0.004) were associated with MMSE below the cutoff points, while DM (*p* = 0.609) presented no association (Table 2 and 3).

**Conclusion:**

In this study, DM was not associated with cognitive ability when compared to APOE4 allele, education, and age. These are our preliminary results. Further studies with a larger sample size are needed to deeply analyze the interaction of DM with other variables strongly linked to cognitive ability and dementia diagnosis.